# Wastewater Treatment Plants as a Source of Malodorous Substances Hazardous to Health, Including a Case Study from Poland

**DOI:** 10.3390/ijerph20075379

**Published:** 2023-04-03

**Authors:** Joanna Czarnota, Adam Masłoń, Rebeka Pajura

**Affiliations:** Department of Environmental Engineering and Chemistry, Rzeszow University of Technology, Powstańców Warszawy 6, 35-959 Rzeszow, Poland; amaslon@prz.edu.pl (A.M.); r.pajura@prz.edu.pl (R.P.)

**Keywords:** hydrogen sulphide, ammonia, volatile organic compounds, impact of odorous substances on humans and the environment, odour nuisance of wastewater treatment plants

## Abstract

Using Poland as an example, it was shown that 41.6% of the requests for intervention in 2016–2021 by Environmental Protection Inspections were related to odour nuisance. Further analysis of the statistical data confirmed that approximately 5.4% of wastewater treatment plants in the group of municipal facilities were subject to complaints. Detailed identification of the subject of odour nuisance at wastewater treatment plants identified hydrogen sulphide (H_2_S), ammonia (NH_3_) and volatile organic compounds (VOCs) as the most common malodorous substances within these facilities. Moreover, the concentrations of hydrogen sulphide and ammonia exceed the reference values for some substances in the air (0.02 mg/m^3^ for H_2_S and 0.4 mg/m^3^ for NH_3_). A thorough assessment of the properties of these substances made it clear that even in small concentrations they have a negative impact on the human body and the environment, and their degree of nuisance is described as high. In the two WWTPs analysed in Poland (WWTP 1 and WWTP 2), hydrogen sulphide concentrations were in the range of 0–41.86 mg/m^3^ (Long-Term Exposure Limit for H_2_S is 7.0 mg/m^3^), ammonia 0–1.43 mg/m^3^ and VOCs 0.60–134.79 ppm. The values recognised for H_2_S cause lacrimation, coughing, olfactory impairment, psychomotor agitation, and swelling of the cornea with photophobia. Recognition of the methods used in practice at WWTPs to reduce and control malodorous emissions indicates the possibility of protecting the environment and human health, but these solutions are ignored in most facilities due to the lack of requirements specified in legislation.

## 1. Introduction

Currently, there is an increase in public complaints about the odour nuisance of municipal management facilities, including wastewater treatment plants [1,2,3]. Wastewater treatment involves a number of physical, biological and chemical processes, which results in the chemical composition of the wastewater varying at different stages of treatment; moreover, it may also vary on a daily basis due to the delivery of wastewater to the treatment plant by slurry trucks or wastewater from various industries (e.g., poultry farms, distillery industry, slaughterhouses, etc.) [4]. Unpleasant odours emitted by wastewater treatment plants have been a serious and growing problem for many years, especially in densely populated local communities. Importantly, in addition to being a nuisance and a health hazard for residents settled in the vicinity of treatment plants, the release of unpleasant odours is also associated with the formation of photo-chemical smog and secondary particulate emissions into the environment [1,2]. Odours released from wastewater treatment plants can cause health problems and often deterioration of neighbourhood and community relations. It should be noted that odours have only recently been recognised as air pollutants, and the biological control of odours and bioaerosols in wastewater treatment plants has received more attention in recent years [5].

Management strategies to reduce odour nuisance include monitoring, evaluation, and control of the substances produced. However, this issue is difficult because odours are complex mixtures of gases that can occur in low concentrations under ambient conditions, and they exhibit high variability over time, which can depend on the weather conditions and wastewater load characteristics. In addition, each odour is characterised by specific cues that include the type of odour, its hedonic quality and intensity [3]. Malodorous emissions from wastewater treatment plants depend on several factors, which include atmospheric pressure, the presence of oxygen, air turbulence above the source, the size of the treatment plant, the technological solutions used, the amount, character, pH and temperature of the wastewater flowing into or delivered to the treatment plant, and the proper operation of the facility [6,7]. According to the study, smell emissions show seasonal fluctuations, and the ambient temperature is closely correlated with the level of these emissions; it is observed that they increase significantly during the summer season, when the temperature is higher [8]. Odour emissions may also be affected by accidental and ad hoc activities of the population related to wastewater disposal [3]. In a study by Mohsen Asadi and Kerry McPhedran [9], the most important parameters affecting the variability of liquid gas and odour emissions from wastewater treatment plants were identified. These included temperature, hydraulic retention time, dissolved oxygen, chemical or biological oxygen demand, and total Kjeldahl nitrogen or total nitrogen concentration. Evaluating greenhouse gas emissions and odours is a key aspect of wastewater treatment plant management and is necessary to identify volatile gas emissions, especially from open facilities, and to determine whether appropriate measures should be taken to deodorise [9]. In addition, such an assessment done in a timely manner will avoid complaints and ensure environmental protection [10].

This article presents the issues related to the formation of odorous substances in wastewater treatment plants, identifying these substances and determining their impact on human health and the environment, indicating especially odorous devices, as well as the possibility of reducing and eliminating emissions from such devices. Stressing that the legitimacy of the subject matter undertaken is due to the lack of existing legal actions in many European Union countries, including Poland, which would directly regulate issues related to the emission (including from wastewater treatment plants) of odorous gases into the environment, the review of knowledge was supplemented by an analysis of statistical data on complaints of odorous nuisance in Poland in 2016–2021 and an assessment of air quality at two selected wastewater treatment plants in Poland.

## 2. Wastewater Treatment Plant as a Source of Malodorous Substances Hazardous to Health

Wastewater treatment plants, due to the processes carried out in them, are a significant source of offensive odours, so for years they have been located on the outskirts of cities to reduce their negative impact on people. Facilities of this type always occupy a large area, usually from a few to several hectares, which is mainly due to technological reasons. The area occupied by small treatment plants is relatively large, but the range of smell impact does not exceed about 200 metres [11]. However, it should be borne in mind that the extent of the impact of such odour is influenced by meteorological and topographical conditions [4]. Unfortunately, the rapid growth of urban agglomerations in recent decades and the lack of restrictive regulations regarding the issuance of permits for residential construction in areas subject to odour nuisance has resulted in a sharp increase in the number of complaints and objections regarding the occurrence of odours [4,11].

The problem of unpleasant odours associated with wastewater treatment begins at the stage of bringing wastewater to the treatment plant through the sewer system and its components (zone I) [2,12,13,14,15].

**Figure 1:**
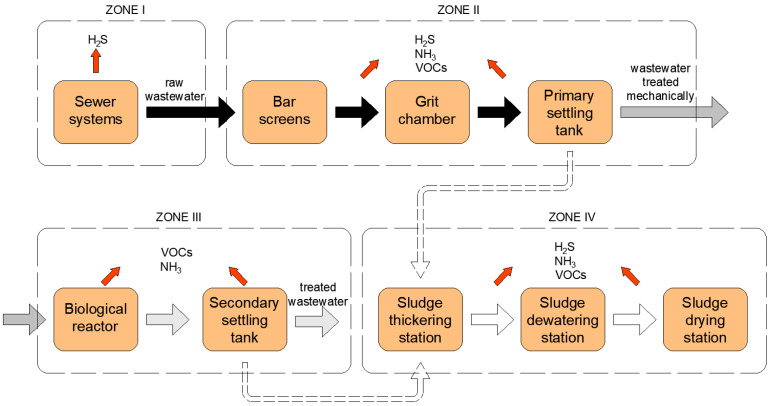
Emission zones at the WWTPs and types of hazardous substances to health emitted from them (own elaboration).

At the wastewater treatment plant itself, two process lines (wastewater and sludge) are distinguished, within which three more zones can be separated, where odours hazardous to health are emitted (Figure 1) [4].

**Figure 2:**
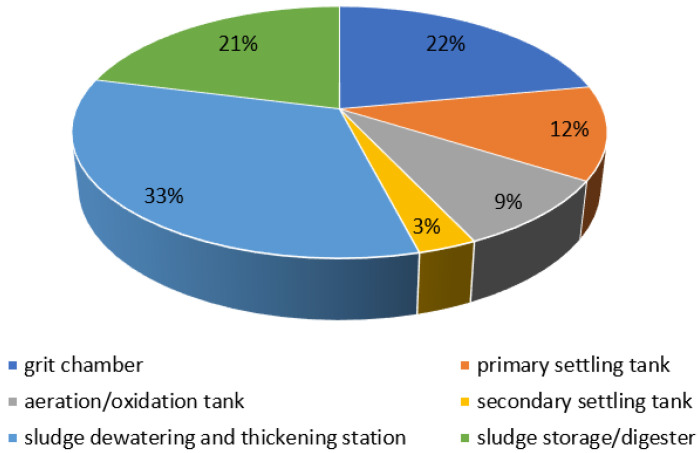
Average percentage distribution of odour emission sources from individual WWTP equipment (own elaboration based on [17]).

Zone one equipment, i.e., pumping stations, waste disposal points, sump pits and sewer lines, are points where wastewater can rot, resulting in the formation of odorants (mainly hydrogen sulphide is formed) [12,13,14]. During the processes carried out in the equipment of zone two (they are supposed to ensure the removal of solids, mineral fines and fractions of oils and fats), many odour compounds are generated, especially hydrogen sulphide. According to the literature, the biggest contributor to odour nuisance in the mechanical part of the wastewater treatment plant is characterised by raw wastewater and the first devices of mechanical wastewater treatment, i.e., grids and screens [4]. The odour nuisance of these facilities intensifies, especially during the summer heat waves, despite the use of hermetisation. A method of reducing odour nuisance in this case is regular replacement of these screens. Grit chambers are characterised by a slightly lower odour nuisance—the sand they dispose of is characterised by about 5% organic fraction content (including PAHs and PCBs) [4]. Primary settling tanks are designed to deprive wastewater of fat and oil fractions and easily sedimentable organic parts. At this stage of wastewater treatment, no mechanical separation of solids is carried out, resulting in lower odour emissions, however, a dross, consisting mainly of fats, collects on the surface of the settling tank, due to which an increased release of volatile fatty acids is observed [4,16]. Senatore et al. [17] reports that the average percentage distribution of odour emissions from grit chambers and primary settling tanks is 22% and 12%, respectively (Figure 2).

In biological reactors, which qualify as the third zone, there are numerous metabolic processes that take place under various conditions (aerobic and anaerobic) in the presence of microorganisms. These processes are often accompanied by the release of odorous compounds, mainly ammonia and hydrogen sulphide [18]. Their emissions can be reduced by bacterial strains that have the ability to neutralise selected groups of odorous compounds [19]. After the activated sludge chambers, the stream of wastewater and biological sludge is directed to secondary settling tanks, where they are separated. This stage is generally characterised by low emissions of odour nuisance compounds [7]. Senatore et al. [17] also indicated that the biological part of the treatment plant shows the lowest average odour emissions—the share of the aerated part and secondary settling tanks as emission sources is 9 and 3%, respectively (Figure 2).

Equipment included in the fourth zone, including gravity thickeners, aerobic stabilisation chambers, separate digesters, dewatering equipment, and station facilities, is characterised by significant odour emissions. Thickening, dewatering, stabilisation, and hygienisation processes are identified as a significant and the most serious source of odour release from municipal management facilities. According to a study by Gębicki et al. [20], almost 37% of the distribution of odour emissions from wastewater treatment plants comes from sludge treatment processes. The thickening of sewage sludge produces volatile fatty acids. In turn, nuisance odour in the hygienisation process is caused by high ammonia emissions. For the hygienisation process, quicklime (CaO) or slaked Ca(OH)_2_ is used, and its oxide form (CaO), after the formation of calcium sulphate, results in the effective reduction of sulphur-containing odour emissions [21]. During the stabilisation process, the amount of organic matter is reduced as aerobic or anaerobic decomposition takes place, the overall level of microorganisms increases, and pathogenic microorganisms are reduced. As a result of this process, the amount of substances hazardous to the environment is reduced, including odour-causing compounds. Different approaches to technical solutions in wastewater treatment plants in terms of the conditions of the stabilisation process can affect the different contents of odorous substances in sludge after the stabilisation process [22]. According to Senatore et al. [17], the sludge section is the main source of odour emissions. The average percentage distribution of odour emissions is 21% for the sludge storage station, while as much as 33% is attributed to the sludge dewatering and thickening station (Figure 2).

In summary, the odour nuisance of wastewater treatment plants can be caused by the introduction of odorants into the treatment plant, as well as their release into the air during the operation of individual facilities of the process line. Odours can also be created by biological activity in the wastewater [23]. Anaerobic processes produce chemical compounds such as hydrogen sulphide, methane, and ammonia, as well as volatile organic compounds such as mercaptans, amines, indole, and skatol. All these substances generate odours that often resemble the smell of rotten eggs, ammonia or garlic, but are also described as organic or earthy [24].

## 3. Effects of Malodorous Substances on Human Health and the Environment

Among the molecules “produced” in wastewater treatment plants and responsible for unpleasant olfactory sensations are: sulphur compounds (e.g., hydrogen sulphide, thiols), nitrogen (ammonia, amines), as well as other organic compounds (aldehydes, ketones, aliphatic and aromatic compounds). Table 1 summarises the most common odorants along with their odour characteristics and an indication of the degree of annoyance [23,25,26,27,28]. As can be seen from the data in Table 1, the odours generated are characterised by a high degree of odour nuisance. Only the degree of odour nuisance of methylamine and butanone was characterised as low. Such mixtures as acetone, ammonia, pyridine, and propionic and acetic acid were characterised as moderately odorous. Nearly half of the listed chemical compounds are characterised by odours corresponding to rotting decomposition of vegetables, while the remaining substances resemble the smell of faecal matter, sweat or rotten eggs.

**Figure 3:**
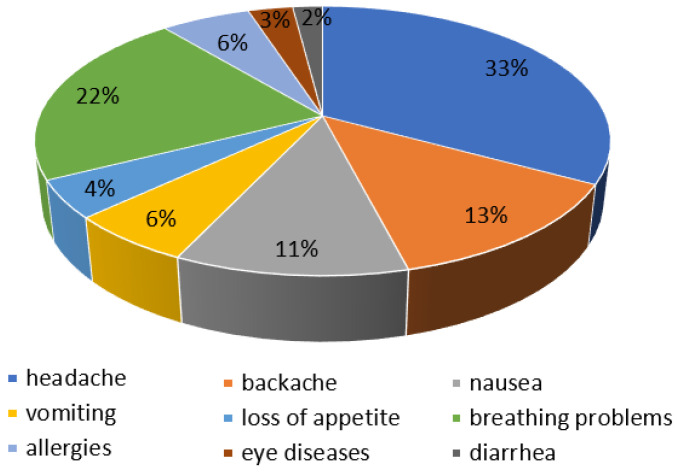
Frequency of physical symptoms with prolonged human exposure to unpleasant odours (own elaboration based on [32]).

Long-term human exposure to odorants can cause negative health effects, beginning with emotional ones, such as anxiety, depression or restlessness, and ending with physical symptoms, which include eye irritation, headache, respiratory problems, nausea and vomiting (Figure 3) [2]. Table 2 shows the characteristics of selected odorants and their effects on the human body and the environment.

Hydrogen sulphide is one of the most dangerous toxic gases that has led to the death of many workers in confined spaces. Hydrogen sulphide poisoning is the second most common cause of death from toxic gases. A fatal case of hydrogen sulphide poisoning in an open space occurred in 2014, when two workers died while opening the hatch of a leachate water tanker. The H_2_S accumulated inside the tanker escaped into the open air after the hatch was opened, and a toxic cloud hung over the tanker, poisoning the workers [39]. Methyl mercaptan, also known as methanethiol, is characterised by a repulsive odour of rotten cabbage, and is absorbed through the respiratory tract. Its effects on the environment and human organisms are not well understood; however, the literature reports a case of human death after exposure to this chemical compound [40]. In addition, in 2014, there was a chemical accident in the United States where there was a release of methyl mercaptan, at which time four workers there died and five were injured [41]. In Poland, according to the Regulation of the Minister of Family, Labour and Social Policy of 12 June 2018 on the highest permissible concentrations and intensities of harmful factors for health in the work environment, substances harmful to health in the working environment are regulated by specifying the Long-Term Exposure Limit (LTEL, in Poland: NDS) and Short-Term Exposure Limit (STEL, in Poland: NDSCh). These concentrations for chemical compounds released from communal management facilities associated with wastewater treatment are summarised in Table 1. In addition, the threshold of detection for each compound is included in the data.

In the literature, hydrogen sulphide and ammonia are the most extensively characterised due to the possible disease symptoms of human contact with the compounds. Concentrations of hydrogen sulphide above 10 mg/m^3^ show negative effects on the human body—there is tearing of the eyes, coughing, impairment of the sense of smell, psychomotor agitation, and swelling of the cornea with photophobia. At concentrations of this gas above 300 mg/m^3^, there is pulmonary oedema and paralysis of the olfactory nerve, which makes the smell of hydrogen sulphide undetectable. Loss of consciousness, respiratory and cardiac disturbances, cyanosis, convulsions, and death are the effects of hydrogen sulphide on humans at concentrations above 750 mg/m^3^. In contrast, concentrations above 1300 mg/m^3^ are equivalent to immediate death [30,35]. In the case of ammonia, concentrations in the range of 300–500 mg/m^3^ irritate the throat and eyes. Fatal poisoning occurs when a person is exposed for 30 min to concentrations in the range of 1750–3150 mg/m^3^. Death within minutes is the result of human exposure to ammonia concentrations in the range of 3500–7000 mg/m^3^ [31]. Negative effects on humans have also been reported for ethanethiol (>10 mg/m^3^, conjunctival irritation, nausea, and headaches appear) and trimethylamine (>48.5 mg/m^3^, coughing, nausea, and redness of the face and eyes appear) [30]. In the case of acetone and acetic acid, on the other hand, the literature gives the lowest concentration of the substance in the air that causes toxic effects in humans over a certain period, or has carcinogenic or harmful effects on foetal development (TCL0). The value is 1210 and 2040 mg/m^3^, respectively.

## 4. Odour Measuring Techniques

With a view to assessing the odour impact resulting from the operation of wastewater treatment plants, two basic techniques for determining odour nuisance can be distinguished. The first is the sensory technique, in which the human sense of smell acts as a detector. It is based on field olfactometry and dynamic olfactometry [42]. Dynamic olfactometry makes it possible to determine the concentration of odour in a sample of air contaminated with malodorous substances, expressed in European odour units per cubic metre (ou_E_/m^3^). A sample is taken into a special bag made of materials resistant to adsorption of the odour compounds [43]. To conduct the analysis, it is necessary to involve evaluators, called panellists. The analysis itself involves diluting the odour sample with neutral air and presenting it in increasing concentrations to the panellists. Since the evaluators are directly sniffing the samples, which potentially contain hazardous and toxic compounds, it is important to keep in mind that adverse body reactions may occur [44,45]. The test continues until panellists begin to smell an odour other than neutral air [45]. A clear limitation of this method is that it does not provide a detailed differentiation of the specific substances responsible for the odour [46]. It is also worth noting that the characteristics of perceived odours are highly subjective, since, depending on the concentration, different people can describe odours quite differently. In addition, the threshold of perceptibility is influenced by the age, gender, and health of the evaluators [47].

Another technique included within the sensory methods is field olfactometry, for which special equipment is used. It involves analysing the air directly at the source and, unlike direct olfactometry, avoids errors due to changes in the composition of the sample during transport and collection in a bag designed for this purpose [14,48]. The use of in situ olfactometers makes it possible to directly estimate the concentration of odour at a given measurement point. The information obtained can be used to verify residents’ complaints about odour nuisance, and furthermore, to assess the hedonic properties of the odours. Field olfactometers find their application in the evaluation of malodorous emissions from wastewater treatment plants, as they are used to assess the intensity of odours in the air within these facilities [49]. The most commonly used device for this is the Nasal Ranger^®^ olfactometer, equipped with two replaceable activated carbon filters, a valve that regulates the intensity of untreated air, an interchangeable mask with valves and a gasket, and a sensor that allows you to control the flow of air through the equipment. It is a relatively small device weighing less than a kilogram [43]. Accurate characterisation of odour emissions is critical to odour management at wastewater treatment facilities, and in order to objectively regulate and monitor odours, they must be quantified [49].

Gases can also be studied by analytical techniques based on the use of gas chromatography or gas sensor arrays [42,50]. The second technique discussed allows qualitative as well as quantitative analysis of the sample under examination for odorous compounds. It is worth noting that the identification and quantification of specific malodorous compounds does not translate directly into the olfactory impression evoked [43,51]. Gas chromatography (GC) is one of the most common techniques used for the determination of odorant compounds. With its help, it is possible to determine the leading substance responsible for the odour response. On the other hand, the combination of gas chromatography together with mass spectrometry (GC/MS) makes it possible to identify the compounds contained in a mixture. This technique is recommended in the vicinity of facilities such as wastewater treatment plants, as it allows the determination of many odourogenic compounds at one time [43,52].

Odour testing cannot be based on only one of the discussed techniques, as both have some drawbacks. The solution to this problem may be a combination of both techniques based on the synergy of their action [50]. The combination of sensory and analytical technology has led to the development of an “eNose” (electronic nose) device equipped with a series of gas sensors and a data processing system. The advantage of this device is its ability to detect both simple and complex odours continuously [10]. According to a study conducted by Lamagna and Lieberzeit [53,54], the use of the “eNose” shows promising results in classifying odours present in the air, but also in water. The static “eNose” has found application in monitoring individual points of facilities such as landfills, farms, ports and composting plants. However, they are inadequate for assessing odour events at sites such as wastewater treatment plants because of the many outbreaks that emit odours as well as the area occupied by them (settling tanks, bioreactors, anaerobic digesters). Monitoring such objects from a single location is difficult and the results can be unreliable. One possible solution to this problem is a “sniffing drone”. Burgues [18] suggests using an “eNose” mounted on a drone for obtaining spatial measurements from wastewater treatment plants. To take a measurement, this device flies to the selected measurement location, where it remains hovering for about 5 min, during which time it takes continuous measurements that allow the variability of emissions from the source under study to be assessed. This innovative method represents an important step in the aspect of measuring odour emissions from wastewater treatment plants and can also contribute to improving the quality of life for people living near these facilities [18].

## 5. Evaluation of the Problem of Odour Nuisance of Wastewater Treatment Plants on the Example of Poland

### 5.1. Analysis of Statistical Data on Odour Nuisance Complaints with Consideration of Wastewater Treatment Plants as Municipal Facilities

**Figure 4:**
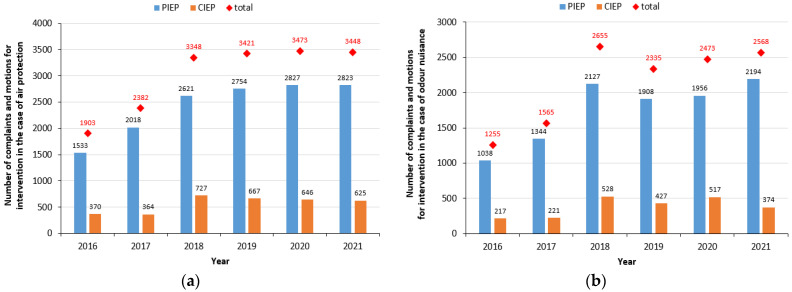
Number of complaints and requests investigated by PIEP and CIEP in the field of: (**a**) air protection; (**b**) odour nuisance (own elaboration based on [55]).

Currently in Poland, complaints and requests for intervention in the field of air protection and odour nuisance can be submitted at the provincial level to the Provincial Inspectorate of Environmental Protection (PIEP) and at the national level to the Chief Inspectorate of Environmental Protection (CIEP). An analysis of data from 2016 to 2021 covering the number of all complaints and requests for intervention by one of the above-mentioned Environmental Inspectorates shows that the number of handled cases for both air protection and odour nuisance is increasing from year to year (Figure 4).

It can also be observed from the data that the total number of air protection cases investigated increased by 479 (more than 25%) from 2016 to 2017, while an increase in the total number of complaints by 1545 (more than 80%) can be seen between 2016 and 2021. Looking at the statistics of odour nuisance cases, it is worth pointing out that the highest increase was from 2017 to 2018—1090 more applications were investigated (almost 70%). The difference between the number of all odour nuisance applications investigated in 2016 and in 2021 is 1313 (more than 104%). The above data clearly indicate that Polish society is reacting to adverse events occurring in the environment, which can affect people’s health and living comfort. This is the result of raising people’s awareness through various training and government programs aimed at bringing the impact of air quality on people’s quality and comfort of life closer.

The two institutions (PIEP and CIEP) together investigated a total of 30,826 applications in the years under review, of which as many as 41.6% were related to odour nuisance. In terms of air protection, PIEP investigated 14,576 applications, accounting for 81%, while CIEP investigated 3399 (19%) (Figure 4a). In the case of odour nuisance cases, the largest number of complaints were also filed at the provincial level—PIEP investigated 10,567 applications (about 82%), while CIEP accepted 2284 applications (Figure 4b). Community Reaction (CR), determined based on the collected and analysed complaints from the population, is one of the methods used in European countries to assess the degree of odour nuisance in a selected area [56]. Analysis within the country of the number of applications that constitute complaints about odour nuisance makes it possible to identify areas in, for example, a voivodeship that are prone to odour nuisance. Moreover, such complaints also make it possible to identify the type of sources that emit substances that contribute to the deterioration of odorous air quality [56].

**Figure 5:**
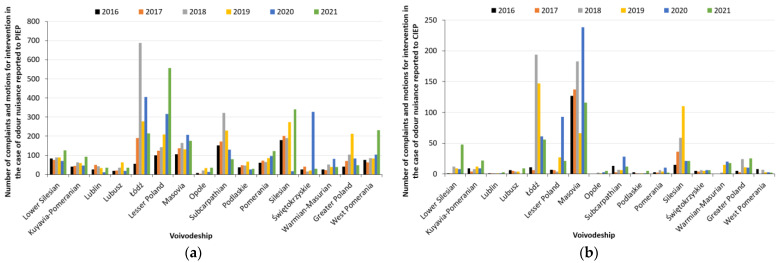
Summary of number of complaints to PIEP (**a**) and CIEP (**b**) about odour nuisance by voivodeship (own elaboration based on [55]).

The number of complaints to PIEP and CIEP about odour nuisance by voivodeship in Poland is shown in Figure 5a,b. The largest number of odour nuisance complaints in the years analysed was recorded for the following voivodeships: Łódź (2305), Masovia (1789), Lesser Poland (1607), Silesia (1464) and Subcarpathia (1156), which are ranked 6th, 1st, 4th, 2nd, and 8th in the placement of voivodeships by population. In contrast, the fewest complaints were recorded in the Opole (only 127), Lublin (209), Lubusz (220) and Podlasie (264) voivodeships. Residents of the Masovian Voivodeship most often address complaints to CIEP. In the case of the Lodz Voivodeship, both environmental institutions are recipients of complaints. At the voivodeship level (to PIEP), most complaints are received in the provinces of Lesser Poland, Subcarpathia and Silesia. According to Sówka [56], the identification of complaints on an area-by-area basis within a country allows, for example, the identification of areas for which it is possible to carry out an assessment of the likelihood and magnitude of the occurrence of an odour nuisance according to the GIS (Geographic Information System) technique.

**Figure 6:**
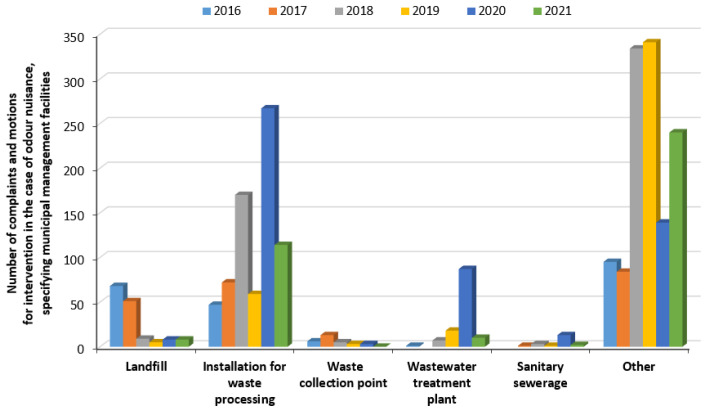
Number of notifications between 2016 and 2021 for odour nuisance of municipal management facilities (own elaboration based on [55]).

In the applications reported in the years under review, from the group of municipal management facilities, landfills, waste treatment facilities, waste collection points, wastewater treatment plants and sewer systems were indicated as odour nuisance facilities. The largest number of complaints were related to waste treatment facilities (729 out of 2284 motions). However, landfills and wastewater treatment plants were also frequently indicated, with the number of applications indicating these facilities, respectively, being 149 and 123 out of the 2284 motions (Figure 6). Attention should also be paid to the “other” group included in Figure 6, especially since 1233 odour nuisance complaints are included in it. The data provided by the environmental institution did not indicate whether these complaints relate directly to the wastewater and waste industry. Research studies have already indicated that the system of recording odour nuisance complaints in Poland requires determining the level of detail of the data collected [56]. A solution to this issue could be the development of a standard odour nuisance complaint card, in which the source (even a potential one) of malodorous emissions would have to be indicated during the report.

**Figure 7:**
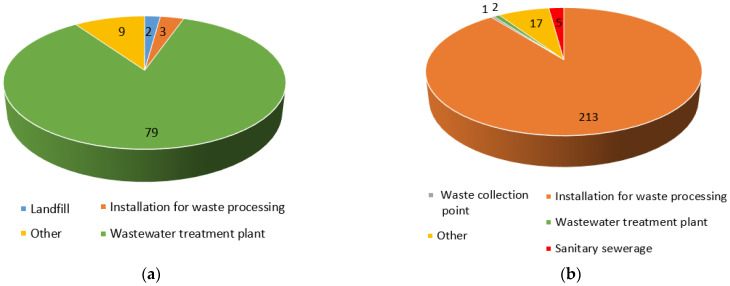
Number of complaints from 2020 about odour nuisance of wastewater treatment plants compared to other municipal facilities: (**a**) in the Lesser Poland Voivodeship; (**b**) in the Masovian Voivodeship (own elaboration based on [55]).

Complaints about odour nuisance at wastewater treatment plants vary widely by province within Poland. For example, in 2020, there were 79 complaints related to odour nuisance of wastewater treatment facilities in the Lesser Poland province, accounting for as much as 85% of complaints targeting municipal facilities (Figure 7a). In contrast, the Masovian Voivodeship showed a negligible share of complaints against wastewater treatment plants, amounting to about 0.8% (2 complaints out of 238) (Figure 7b).

**Figure 8:**
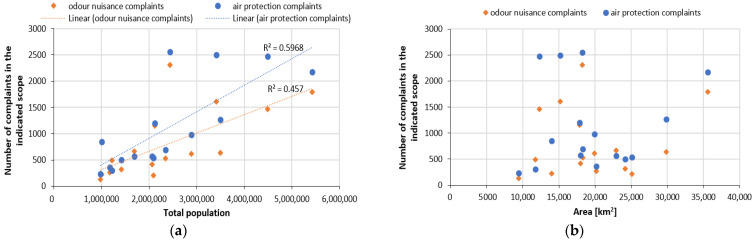
Number of complaints and applications handled in the field of air protection and odour nuisance in relation to: (**a**) total population of the voivodeship; (**b**) area of the voivodeship.

An examination of the relationship between the number of complaints in a specific range and the total population in a given area (voivodeship territory) shows that an approx. 46% change in the number of residents explains the variation in the number of odour nuisance complaints, and an approx. 60% change in the number of residents explains the variation in the number of air protection applications (Figure 8a). Relating the number of complaints to the area of each voivodeship, there was no relationship between these variables (Figure 8b). The voivodeship with the highest number of odour nuisance complaints (Łódź) ranks only 9th (out of 16) in the placement of voivodeships by area. The results obtained allow us to conclude that there are other factors that affect the variability of the number of complaints, such as the degree of development, industrialisation, etc.

### 5.2. Recognition of Odour Nuisance and Air Quality Due to Malodorous Substances

#### 5.2.1. Odour Nuisance of Polish Wastewater Treatment Plants

In Poland, a dozen years ago, odour nuisance diagnosis was carried out for a total of 462 wastewater treatment plants, including 423 plants described as medium and 39 plants described as large [57].

The investigation showed that the bar screens and primary settling tanks are the main sources of odour nuisance of the mechanical part in large- and medium-sized wastewater treatment plants. The analysis of the data presented showed that grilles were used in all large wastewater treatment plants, and 56.4% of closed units and as many as 75.0% of open units were indicated as facilities exhibiting odour nuisance. In the case of medium-sized WWTPs, grilles appeared in 91% of the WWTPs, of which 33.9% of closed units and 51.3% of open units were characterised by odour nuisance. Nearly 85.0% of large wastewater treatment plants opted for primary settling tanks, of which as many as 66.7% of the open primary settling tanks were rated negatively due to odour. It is worth noting that there were only three closed primary settling tanks and none of them emitted odour compounds, which means that the total number of odour nuisance devices were open primary settling tanks. An analysis of average wastewater treatment plants shows that primary settling tanks were the cause of odour nuisance for 36.7% of the facilities (17.5% of closed system equipment and 42.2% of open system equipment). From the reconnaissance carried out, it appears that for all facilities of the mechanical part, facilities operating as open are responsible for more odour nuisance [29,57,58].

Recognition of the odour nuisance of Polish facilities confirms that the biological part is characterised by relatively low odour nuisance, and usually the number of nuisance facilities from this zone of the wastewater treatment plant does not exceed 21% of all facilities. The anaerobic chamber within large wastewater treatment plants was indicated as the most onerous in terms of malodorous emissions (about 57% of facilities). It is worth noting that emissions of malodorous compounds were recorded for facilities operating in an open system [29,57].

The sludge section is the most problematic zone in terms of odour nuisance, which results from the presence of sludge thickening, sludge stabilisation and sludge dewatering devices. According to a study by Szynkowska and Zwoździak, the percentage of treatment plants where thickeners are a source of odour nuisance ranges from 23.1% to 37.1% for large treatment plants and from 28.0% to 30.6% for plants described as medium. As for the mechanical-biological part, closed facilities are characterised by lower odour nuisance. More than 35% of the open separate aerobic chambers present at medium-sized wastewater treatment plants are rated negatively due to malodorous substances. On the other hand, in the case of separated closed oxygen chambers, odour emissions affected only 22.3% of the facilities. It was also noted that separated digesters had the lowest odour nuisance compared to other stabilisation facilities. It should be noted that 100% of the composting facilities present at large WWTPs in Poland were sources of odour nuisance. In addition, dewatering facilities were characterised by high values (usually more than 50% of the facilities in the group). In contrast, relatively low odour nuisance was recognised in hygienisation facilities, as malodorous odours affected a maximum of 24.1% of facilities [57,58].

#### 5.2.2. Assessment of Air Quality Due to the Presence of Hydrogen Sulphide, Ammonia and Volatile Organic Compounds

**Figure 9:**
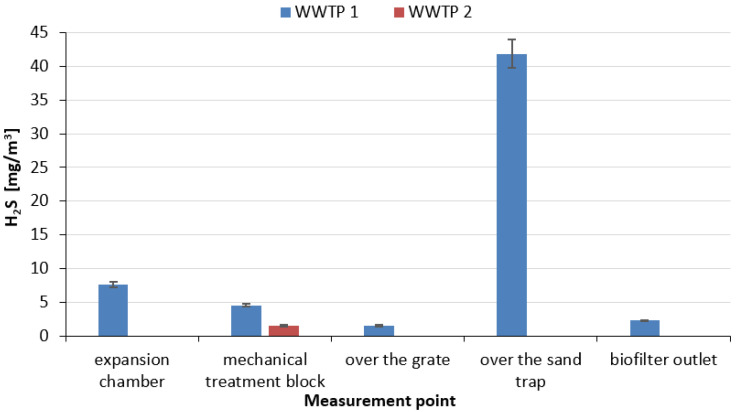
Concentration of hydrogen sulphide in WWTP 1 and WWTP 2 (Poland) at different measurement points (own elaboration based on [60]).

An analysis of data for two wastewater treatment plants operating with MBBR (Moving Bed Biofilm Reactor) technology, the first with a throughput of 1200 m^3^/d (WWTP 1), the second with a throughput of 2800 m^3^/d (detailed characteristics of this wastewater treatment plant in [59]) (WWTP 2), provided by one of the Water and Wastewater Management Plants in the Subcarpathian Voivodeship (Poland), made it possible to identify the concentrations of selected malodorous substances, i.e., hydrogen sulphide (measurement of H_2_S using a Keison infrared gas analyser type Geotech GA 2000; detection rate 0–500 ppm; measurement error ± 10%), ammonia (measurement of NH_3_ using a multi-parameter portable gas analyser from OMC ENVAG type Gasmet DX-4000; detection rate < 1 ppm, measurement error < 2%) and volatile organic compounds (measurement of VOCs using a portable volatile organic compound analyser from J.U.M. model OVF-3000 J.U.M) at various locations in the treatment plant. Measurements at the treatment plants were conducted in windless weather and at an air temperature of 21 °C. In the case of WWTP 1, the highest concentration of hydrogen sulphide was found above the grit chamber, which is a self-contained open facility (41.86 mg/m^3^), but facilities such as the expansion chamber (facility closed) and the mechanical treatment block (closed technological hall) were also characterised by high emissions of this odorous substance of 7.61 mg/m^3^ and 4.57 mg/m^3^, respectively (Figure 9). In contrast, for WWTP 2, the mechanical treatment block (closed technological hall) was the single point from which hydrogen sulphide release took place (1.52 mg/m^3^). It should be noted that in the case of WWTP 2, mechanical treatment is carried out using an interlocked Huber Rotamat Ro5-HD unit, which is a fully enclosed unit and is a combination of a screen and grit chamber. In both WWTPs, air drawn from the expansion chamber, and from above the open grit chamber in the case of WWTP 1 and from the closed screen-grit chamber in the case of WWTP 2, is directed to the biofilter. In both treatment plants, hydrogen sulphide concentrations were not recorded in the biological wastewater treatment section (MBBR reactors) and in the sludge treatment section (sludge lagoon). According to the data in Table 1, the measured values at some points exceed the maximum permissible concentration (7 mg/m^3^). It is worth noting that the reference value for hydrogen sulphide in the air (averaged over a 1 h period), in accordance with the Regulation of the Minister of the Environment of 26 January 2010 on reference values for certain substances in the air, was set at 0.02 mg/m^3^.

Relating the above values to literature data for a 570,000 m^3^/d wastewater treatment plant (Kuwait), where hydrogen sulphide concentrations in 2013–2015 at the main pumping station oscillated in the range of 2.29 mg/m^3^ to 25.09 mg/m^3^ [61], one can point to a significant problem in hydrogen sulphide release at the assessed treatment plants. For comparison, the average annual content of hydrogen sulphide in the air above the settling tank at one Polish wastewater treatment plant with a capacity of 200,000 m^3^/d (Koziegłowy, measurements carried out in 1995–2010) was 0.9 µg/m^3^, a value below the reference value and the threshold concentration, and yet residents of the surrounding area felt the odour nuisance of this treatment plant [62]. It should be borne in mind that the measurements at the two Polish WWTPs were conducted during the summer, and higher ambient temperatures increase the activity of sulphur-reducing bacteria, resulting in higher H_2_S concentrations. Hamoda and Alshalahi [61] evaluated the variation of the hydrogen sulphide concentration in relation to temperature in one year of wastewater treatment plant operation (2016) and showed that the lowest H_2_S concentration of 0.98 mg/m^3^ occurred at 30 °C, and the highest concentration of 13.80 mg/m^3^ at 41 °C. In WWTP wastewater delivery systems, hydrogen sulphide can accumulate to very high levels (up to 1115.089 mg/m^3^), causing unpleasant odour, corrosion, but most importantly, a threat to human health and safety [15]. Literature data on hydrogen sulphide concentrations in the mechanical part of wastewater treatment plants (sometimes distinguishing between individual units) vary widely. For example, in one Greek WWTP, the measured hydrogen sulphide concentrations oscillated in the range of 0.030–3.966 mg/m^3^ for screens, 0.007–0.103 mg/m^3^ for grit chambers and 0.001–0.024 mg/m^3^ for the primary settling tank [63]. At one Dutch WWTP operating with BNR (Biological Nutrient Removal) technology, hydrogen sulphide concentrations in the first stages of mechanical wastewater treatment averaged only 0.092 mg/m^3^ [64]. On the other hand, from a German WWTP, the maximum detected concentration of hydrogen disulphide (decomposes into hydrogen sulphide and elemental sulphur) on the screens was 6.54 mg/m^3^ [65]. A study conducted in Beijing for a treatment plant operating with SBR (Sequencing Batch Reactor) technology showed that hydrogen sulphide was one of the main compounds emitted during the first stage of treatment [66]. The authors emphasise the strong correlation of hydrogen sulphide emission flux with the temperature, as higher values were achieved in the spring (3.9 mg S/m^2^·h) and summer (4.0 mg S/m^2^·h) seasons than in the autumn (2.5 mg S/m^2^·h) and winter (1.5 mg S/m^2^·h) seasons [66]. As mentioned earlier, in both WWTP 1, and WWTP 2, hydrogen sulphide concentrations were not recorded in the biological treatment section of the wastewater treatment plant and in the sludge section. According to the literature, hydrogen sulphide concentrations in biological reactors are relatively low (0.0001–0.023 mg/m^3^), with the most common being at the level of 0.0001–0.045 mg/m^3^ [63,65,67,68]. The sludge section shows wide variation in hydrogen sulphide release. For example, the hydrogen disulphide concentration for the sludge thickener is 3.22 mg/m^3^, and for the centrifuge is 14.02 mg/m^3^ [65]. In a study by Lasaridi et al. [63], the measurement point was the dewatering presses, and the measured H_2_S concentrations ranged from 0.010 to 0.125 mg/m^3^. On the other hand, for the sediment plots, hydrogen sulphide concentrations were recorded at a level of 0.043 mg/m^3^ [67]. Air quality studies at the wastewater treatment plant in Łask (Poland), which has a capacity of 6000 m^3^/d, showed that the permissible values of pollutant concentrations in the air were exceeded. The highest concentrations of hydrogen sulphide were recorded near the administration building (0.151 mg/m^3^), near the digesters (0.126 mg/m^3^) and near the sludge dryer (0.117 mg/m^3^). Slightly lower concentrations were recorded near the sludge plots (0.099 mg/m^3^) and the sewage pumping station (0.062 mg/m^3^). Importantly, at a measurement point near which there was no technological equipment, the concentration of this gas was 0.012 mg/m^3^ [69].

**Figure 10:**
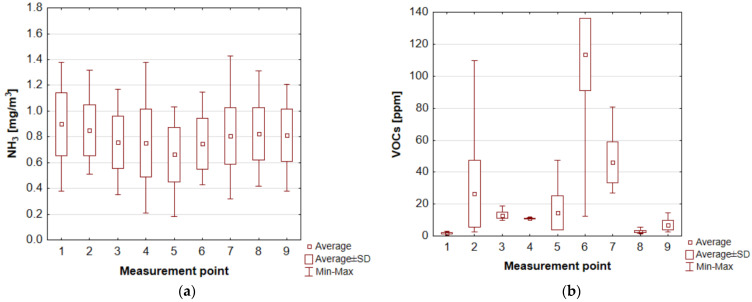
Concentration values at different measurement points of WWTP 1: for (**a**) ammonia; (**b**) volatile organic compounds (own elaboration based on [60]). 1—entrance to the wastewater treatment plant, 2—expansion chamber, 3—mechanical treatment block, 4—measurement directly over the screen, 5—measurement directly over the grit chamber, 6—outlet from the biofilter, 7—biological treatment block, 8—sludge lagoon 1 (aeration off), 9—sludge lagoon 2 (aeration on).

**Figure 11:**
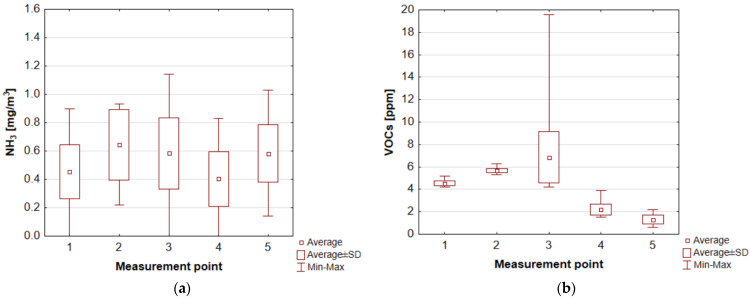
Concentration values at different measurement points of WWTP 2: for (**a**) ammonia; (**b**) volatile organic compounds (own elaboration based on [60]). 1—entrance to the wastewater treatment plant, 2—mechanical treatment block, 3—biological treatment block, 4—sludge lagoon (aeration on), 5—container with screenings.

The ammonia concentrations at WWTP 1 measurement points varied from 0.18 to 1.43 mg/m^3^, with average values oscillating from 0.66 mg/m^3^ (over an open grit chamber) to 0.90 mg/m^3^ (entry to the treatment plant) (Figure 10a). At WWTP 2, the average values for ammonia concentrations ranged from 0.40 to 0.64 mg/m^3^, with the highest values recorded at the mechanical (0.93 mg/m^3^) and biological treatment blocks (1.14 mg/m^3^) and at the screening’s storage point (closed container) (1.03 mg/m^3^) (Figure 11a). The reference value for ammonia in the air (averaged over a 1 h period), as specified in the Regulation of the Minister of the Environment of 26 January 2010 on reference values for certain substances in the air, is 0.4 mg/m^3^.

Huanyu et al. showed that ammonia is one of the main odorous substances in the wastewater pretreatment section (0.25 mg/m^3^), biological reactor (0.44 mg/m^3^), secondary settling tank (0.15 mg/m^3^) and sludge dewatering station (0.47 mg/m^3^). According to a study by Jeon’a et al. [68], NH_3_ concentrations also fluctuate seasonally. The authors showed that ammonia concentrations from the pre-settlement perimeter averaged 0.28 mg/m^3^ in summer and about 0.58 mg/m^3^ in winter. The aerated tanks (reactors), on the other hand, had an average ammonia concentration of 0.13 mg/m^3^ in the summer season and 0.10 mg/m^3^ in the winter season. For the secondary settling tank, the average concentration was 0.12 mg/m^3^ in the summer season and 0.064 mg/m^3^ in the winter season [68]. In a Polish wastewater treatment plant (Koziegłowy), the average annual concentration (from 1995 to 2010) of this gas in the air within the primary settling tank was 1.33 µg/m^3^ [62]. The ammonia content in the air at the aforementioned wastewater treatment plant in Łask (Poland) varied. The highest concentrations—equal to 0.141, 0.129 and 0.126 mg/m^3^—were recorded in the vicinity of the administration building, sludge dryer and digesters, respectively. The lower concentrations occurred near the sludge plots (0.109 mg/m^3^). At the measurement point near which there was no technological equipment, the concentration of this gas was 0.102 mg/m^3^ [69]. One study determined the ammonia emission rate for individual treatment plant facilities. The results show that the average highest emission rate was recorded in the primary settling tank (approx. 200 g/min), while for the other facilities, this parameter was much lower (grit chamber—26 g/min, raw wastewater receiving tank about 8.67 g/min, screens—6.33 g/min, aeration chamber in MBR reactor—4.5 g/min) [70].

The highest concentrations of volatile organic compounds in WWTP 1, which are one of the “components” of unpleasant odours, were recorded at the biofilter outlet (average 113.57 ppm). High concentrations of this group of substances were also recorded in the air at measurement points 2 and 7 (expansion chamber and biological treatment block), which were, respectively, 26.41 ppm and 46.17 ppm (Figure 10b). In the second plant evaluated, high concentrations of volatile organic compounds were recorded in the air at measurement points 1, 2 and 3 (entrance to the wastewater treatment plant, mechanical treatment block and biological treatment block), which were, respectively, 4.54 ppm, 568 ppm and 6.86 ppm (Figure 11b). González et al. [70] determined the average highest VOC emission rate for the primary settling tank (12 g/h). In other units, this parameter was 11.8 g/h for the raw wastewater receiving tank and 0.8 g/h for the screens. It is worth noting that the authors did not detect VOC emissions in the membrane bioreactor (in the aerated and non-aerated parts). Limited emissions were recorded in the dewatering building. Moreover, a study by González et al. [70] indicates that alkanes are the largest group of VOCs emitted within the wastewater treatment plant facilities. Acetal emissions average about 6%, with a significant increase in the non-aerated portion of the membrane reactor (up to about 25%). The highest emissions of alcohols, ketones and sulphur compounds were recorded in the primary settling tank (about 13%). In turn, the highest emissions of esters were in the aerated part of the MBR. High emissions of aromatic hydrocarbons affected the screens, grit chamber and dewatering station. It is worth noting that VOCs are a broad group of compounds, which further include aldehydes, alkenes, carboxylic acids, ethers or phenols, siloxanes, and terpenes.

**Figure 12:**
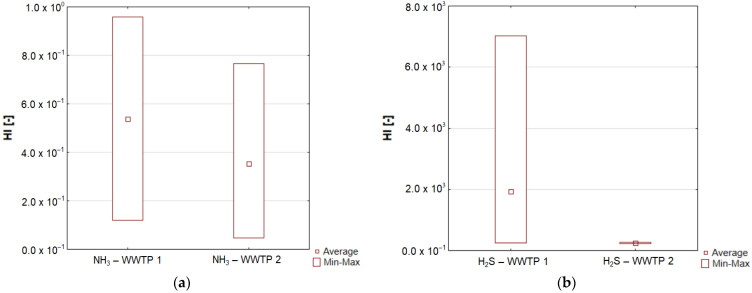
Ranges of hazard index (HI) parameter determined for ammonia (**a**) and hydrogen sulphide (**b**) (HI values were determined by the method proposed by [7], assuming that daily exposure time is 6 h/day, exposure frequency is 350 days/year, exposure duration is 35 years, average time is 25 years for non-carcinogenic risks. According to [71], the reference concentration for NH_3_ and H_2_S was assumed 5.0 × 10^−1^ mg/m^3^ and 2.0 × 10^−3^ mg/m^3^, respectively).

The presence of ammonia, hydrogen sulphide and volatile organic compounds in the air inhaled by the staff of the analysed wastewater treatment plants may pose a health risk to these people. The assessment of this risk was performed in accordance with the recommendations of the US Environmental Protection Agency [71] referring to ammonia and hydrogen sulphide and assuming that the acceptable level of risk of non-carcinogenic compounds is 1.0 × 10^0^ (HI = 1). In the case of VOCs, it is not possible to refer to a specific chemical compound, as the available results were the sum of a group of chemical compounds. HI values, calculated in accordance with the methodology indicated by [7], in the case of ammonia oscillated in the range from 1.2 × 10^−1^ to 9.6 × 10^−1^ for WWTP 1 and from 0.47 × 10^−1^ to 7.7 × 10^−1^ for WWTP 2 (Figure 12a), which clearly indicates that they were not higher than the acceptable level of risk. In turn, the HI values calculated for hydrogen sulphide exceeded the acceptable level, as they varied from 0.26 × 10^3^ to 7.0 × 10^3^ for WWTP 1 and from 0.23 × 10^3^ to 0.28 × 10^3^ for WWTP 2 (Figure 12b). Biliński et al. [7] states that a reliable assessment of the health risk of wastewater treatment plant employees, taking into account the emission of odorous substances, can be obtained by constant monitoring of the atmospheric air in terms of individual chemical compounds emitted in the processes of mechanical and biological wastewater treatment. At the same time, it is necessary to take into account the variable levels of these substances depending on the season and pay attention to such parameters as temperature, solar radiation or relative humidity.

According to information provided by the operator of WWTP 1 and WWTP 2, it appears that it receives numerous complaints from local residents about the odour nuisance of the facilities. Residents usually complain about unpleasant odours in the period from February to June (early spring, spring, early summer). In autumn and winter, such complaints are not received by the operator. Such information confirms that with rising temperatures, pollutants become more volatile, and emissions from surface sources increase. In addition, an increase in air temperature causes an increase in the activity of anaerobic microorganisms in wastewater treatment plants, since the solubility of oxygen in wastewater decreases with the increasing temperature [72]. The operator himself has observed some correlation between the number of complaints and the weather conditions. According to him, he receives the most complaints when there is little wind, no cloud cover and rainless weather. By contrast, in high winds and precipitation, residents do not report air quality problems to him. The literature data confirms that an increase in wind speed results in a decrease in the concentration of the components of the pollution plume, while precipitation results in a decrease in the concentration of ambient air pollutants as a result of their dissipation in water, and in addition, raindrops entrain pollutant particles, transporting them to the ground [73].

Referring to the analysis of the data made available by the operator, as well as to the information provided by him, it should be pointed out that there is a problem of malodorous emissions from these treatment plants. Although such recognition does not make it possible to determine the exact scale of the impact of these facilities on the surroundings, it does provide a signal for action, as it should be remembered that the same concentration of odour may cause different levels of discomfort to different recipients due to different assessments of the source of the odour, sensitivity and degree of activity. In this particular case, which may serve as a good model for action for operators of other wastewater treatment plants in Poland, it is worth considering conducting surveys of local residents of the treatment plant. This type of measure will primarily make it possible to determine the extent of the odour impact of treatment plants. In addition, reference to the opinion of the local community will make it possible to assess the degree of odour nuisance of municipal management facilities in the selected area, taking into account the socio-demographic conditions of the people living in the area. Taking into account the lack of direct legal regulations in Poland, it should be emphasised that, at present, conducting studies related to odorous emissions in and around wastewater treatment plants is at the discretion of the managers of the municipal enterprise, and the reluctance to conduct these studies is often dictated by financial considerations. Actions taken by the operator of WWTP 1 and WWTP 2 (diligent collection of complaints from local residents, commissioning of an expert opinion) allowed him to introduce, as far as financially possible, improvements in the technology at both facilities. One of such actions was a thorough evaluation of the functioning of the installation for capturing air directed to the biofilters and the biofilters themselves, which showed, among other things, the need to replace the natural filtering material with a mineral one, as the original one had also lost its filtering properties, and thus its ability to remove malodorous substances.

## 6. Opportunities to Counteract the Odour Nuisance of Wastewater Treatment Plants

### 6.1. Legal Regulations in Poland to Combat Odour Nuisance

In Poland, there are no legal regulations that directly define standards for combating odour nuisance. It should be emphasised that the European Union also does not have legal regulations that would standardise emissions of malodorous substances that are a nuisance to the environment, and the European Parliament and Council Directive 2008/50/EC on air quality and clean air for Europe, and European Parliament and Council Directive 2010/75/EU on industrial emissions including integrated pollution prevention and control are indicated as documents that relate in some way to odour nuisance [74]. Moreover, in 2016/2017, a petition was sent from Poland to the European Parliament to request an addition to Directive 2008/50/EC, as well as the development of a directive regarding the assessment and management of odour levels in the environment. However, despite awareness of the adverse effects of odour nuisance on people, the European Parliament recognised that odour nuisance is a local problem and it is at this level that it should be eliminated [75].

In Poland, there are a number of national regulations that indirectly touch on the problem of reducing odour nuisance. Selected regulations are included in Table 3. The latter document includes a list of types of installations that may cause significant pollution of individual natural elements or the environment as a whole. The provisions of this regulation exclude activities carried out during the treatment of municipal wastewater from installations related to waste management (group 5) and from the group of other types of installations (group 6), which are likely to significantly affect the pollution of the environment. It should be noted that for the treatment of municipal wastewater, wastewater treatment objectives are legally defined in terms of the quality of the treated wastewater, and the necessary permit to start construction does not include odour emissions from these plants [76].

Poland has also developed a Code against Odour Nuisance [77]. However, this is a document that cannot be the basis for shaping the legal situation of individuals, legal entities, or any other entities. Work on this document made it possible to create a list of substances and chemical compounds that cause odour nuisance, and to determine at what distances from buildings objects (buildings) can be located, the operation of which is associated with the risk of creating odour nuisance (including in the area of animal husbandry and breeding).

At present, few European Union countries have regulations based on air quality standards, however, the problem has been recognised, public awareness is increasing, and in response to the needs of mankind, more and more countries are including in legislation the possibility of preventing odour nuisance and maintaining good air quality. In the absence of unambiguous and generally applicable regulations, European Union countries apply various standards and internal regulations to control malodorous odours. An example is the Netherlands Emission Guidelines (NeR), which sets criteria for odour emissions and emission, but primarily focuses on preventing and reducing odour nuisance [78]. It should be noted that this document is not a legally binding act. The Netherlands also has regulations on odour concentration limits, where the country’s limit is 0.5 ou_E_/m^3^ [56]. Similar regulations are in place in Belgium, where the goal is to reduce the number of people exposed to odour nuisance to zero. Very well-developed legislation on air quality control is respected in Germany. Issued in 1986, the “Law on Air Protection” and the “Technical Instruction on Air Quality Control” specify in detail the substances and their emission limits in terms of human health protection and environmental protection. In addition, the law lays down guidelines to prevent malodorous substances in various technologies, moreover, it specifies the minimum distance from a disturbance in the case of installations prone to emissions of odorous compounds. The Czech Republic, on the other hand, has a regulation on sources of air pollution and sets emission limits, however, it only applies to the agricultural sector [79].

The aforementioned Directive 2010/75/EU of the European Parliament and of the Council covers various industries and specific activities and processes [80]. Unfortunately, it does not include wastewater treatment plants, but only processes related to waste management. The directive only includes provisions for the treatment of wastewater not covered by Directive 91/271/EEC and coming from installations such as waste incineration plants and waste co-incineration, operated by an independent operator. Furthermore, no provisions are included in the Urban Wastewater Treatment Directive 91/271/EEC that would require treatment plant operators to control emissions of malodorous compounds [76]. This means that, despite the common approach to the odour problem, there are no regulations in European countries that relate directly to wastewater treatment facilities. The extent of the impact of the legislation in each country is summarised in Table 4. Australia and New Zealand have strict criteria for odour evaluation, and in addition, odour emissions are regulated by law. However, no criteria are set for odour evaluation, which means that a piggery will be evaluated based on the same odour index as a wastewater treatment plant [81]. Criteria for odour evaluation vary depending on the size of the population that may be affected by odour. In the southern part of Australia, the odour limit ranges from 2 ou_E_/m^3^ to 10 ou_E_/m^3^, the highest values apply to small concentrations of people, while the lowest values (2 ou_E_/m^3^) apply to residential areas of 2000 or more people [56]. China and Japan, on the other hand, set maximum emissions from emitters in addition to emission regulations, due to higher population density. China’s standard sets emission limits for eight odorous substances (hydrogen sulphide, methanethiol, dimethyl sulphide, dimethyl disulphide, carbon disulphide, ammonia, trimethylamine, and styrene) [81]. Most of these substances are emitted from wastewater treatment plant facilities, which in a way requires operators to control emissions and possible ways to prevent odours at wastewater treatment plants. In Hong Kong, the odour concentration limit is 5 ou_E_/m^3^, while in Taiwan the value is 10 times higher [56]. Japan is the only country discussed so far to have an odour regulation standard for odours contained in wastewater, and local authorities have the right to inspect the facilities in question. If the living environment deteriorates, authorities can order improvements in conditions or even impose fines. In addition, Japan has distinguished 22 substances referred to as “repugnant odorants” [82].

### 6.2. Solutions to Reduce and Eliminate the Impact of Malodorous Substances Generated in Wastewater Treatment Plants on People and the Environment

The choice of the appropriate method to reduce the impact of malodorous sub-stances generated in WWTPs (including H_2_S, NH_3_ and VOCs) on the surrounding environment depends on the source and type of emitter/emission. There are definitely more opportunities for the prevention and removal of malodorous odours for point source emitters and systems with encapsulation. For surface emitters and systems where encapsulation is not possible, malodour prevention and removal are more limited.

#### 6.2.1. Hermetisation

Hermetisation greatly facilitates deodorisation and is one of the simplest methods of reducing odour nuisance. In addition to reducing odours, this method plays an important role in reducing the migration of harmful bio-aerosols from wastewater [88,89]. However, this is a costly investment consisting of hermetically covering the facility emitting malodorous compounds, and equipping it with ventilation systems and gas purification equipment. It should be borne in mind that odours should not spread inside hermetically sealed facilities, and their emission into the atmosphere should be limited [62]. According to a study conducted by Murawska and Rauba [88], it is clear that hermetisation is an effective method for reducing the release of malodorous substances [88]. Measures implemented at the Płaszów wastewater treatment plant (Poland) also confirm the positive impact of encapsulating the facilities. The treatment plant located there struggled with odour problems so strong that local residents claimed it “made normal living impossible”. The open sewers and facilities of the mechanical part of the treatment plant were identified as the most troublesome. At that time, the decision was made to hermetise the tributary sewer, the grate station, and the pumping station, which resulted in achieving the intended goal and reducing odour emissions. It is worth noting that filters filled with activated carbon were used to filter the malodorous air [90]. A solution based on covering the primary settling tanks with self-supporting aluminium domes and purifying the air accumulated under these domes in two-stage deodorisation plants with a capacity of 12,000 m^3^/h each (the first stage of air purification is carried out in a mineral bed, while the second consists of dry chemical adsorption) was applied at the Koziegłowy wastewater treatment plant (Poland). The measurable effect of this measure was to reduce odour emissions into the atmosphere, thereby reducing the facility’s impact on the environment and human health. For example, the maximum concentration of H_2_S in the air flowing into the deodorisation plant was about 80 mg/m^3^, and in the outflow from the plant was <0.10 mg/m^3^. At the “Hajdów” wastewater treatment plant (Lublin, Poland), on the other hand, the hermetisation of facilities provided an alternative to demarcating a costly protection zone around the facility. By sealing the most emissive facilities, which included, for example, the grit chamber, the grate hall, and the thickeners, and using biofilters to treat malodorous air, the odour nuisance of the plant was reduced for local residents and employees who supervise the plant’s operation [91]. Similar findings were published in a paper by Brudniak and co-authors [92]. They showed that the odour nuisance of point emitters can be reduced by hermetisation. The authors also point out the high financial burden for encapsulation of surface sources, so it is suggested to use other methods to reduce odour nuisance [92]. It is worth noting that hermetisation should be supported by other processes and methods, which are briefly characterised in the following two sections.

#### 6.2.2. Sorption Processes

Referring to the point emitters and encapsulated systems found in wastewater treatment plants, various methods can be used in practice to counteract nuisance odour emissions. For example, the Seville wastewater treatment plant, which treats wastewater using the activated sludge method, successfully uses an odour deodorisation process using adsorption on activated carbon. The process involves drawing air through fans and directing it to activated carbon filters, where malodorous substances are adsorbed and the clean air is discharged to the atmosphere [93]. Another solution is gas deodorisation based on the absorption method, which uses the ability to dissolve odour-generating compounds in the absorption liquid. The reduction of odour nuisance occurs due to the transport of the mass of the malodorous pollutants from the gas to be treated to the absorbent (liquid). The efficiency and effectiveness of this process is influenced by the contact area between the gas and the liquid, and the degree of solubility of the contaminants [94]. The deodorisation process of air extracted from hermetically sealed objects using the phenomenon of absorption (process AQUILAIR^®^ Veolia Water Technologies) is used, among others, in one of Poland’s wastewater treatment plants (“Czajka” WWTP, Warsaw). The system consists of two counter-current scrubbers connected in series, one acidic and the other alkaline-oxidising. The removal efficiency of malodorous substances for this deodorisation system is up to 98.8% for hydrogen sulphide (with an inlet concentration of up to 51.26 mg/m^3^) and up to 78.9% for ammonia (with an inlet concentration of up to 8.96 mg/m^3^). It is worth mentioning that the project predicted inlet hydrogen sulphide concentrations of 5–10 mg/m^3^ and 8–16 mg/m^3^ of ammonia [95,96].

#### 6.2.3. Biological Methods

On the other hand, a wastewater treatment plant in Iran uses an odour control system with a biological filter, which, under appropriate operating conditions, has a hydrogen sulphide and ammonia removal efficiency of 92% and 99.5%, respectively [97]. Biofiltration and the use of bio-scrubbers are typical biological methods used to dispose of malodorous gases. They are characterised by relatively simple technology and relatively low operating costs. Air purification with a biofilter involves introducing contaminated air into a biological bed so that as much air as possible reaches the bed. Bacteria generated on the surface of the fill absorb and use for their life processes the pollutants contained in the air (including odour-forming substances) [4]. As reported in the literature, biofilters are particularly effective in removing ammonia and hydrogen sulphide from waste gases [98]. The principle of bio-scrubbers is similar and also proceeds in two stages: the first is the absorption of pollutants and the second is their biodegradation through the action of microorganisms [99]. The main difference between biofilters and bio-scrubbers is that the treatment of gases in a bio-scrubber is carried out using two separate devices (absorber + bioreactor) [100]. The periodic removal of excess microorganisms to prevent the overgrowth of the fill is important in this method. Operational data to date indicate that bio-scrubbers are effective in removing odorants containing organic sulphur compounds [101]. A water bio-scrubber has been used successfully in a French wastewater treatment plant, specifically in a digested sludge drying line. The authors emphasise the fact of using cold water in the scrubber, which reduces the operating costs while also increasing the removal of VOCs. According to the study, thanks to the use of a water bio-scrubber and an activated carbon filter, almost 97% efficiency in removing malodorous substances from the sludge dryer was achieved [102].

#### 6.2.4. Thermal Neutralization and Ozonation

A pilot study was conducted at a municipal wastewater treatment plant in Turkey in the application of ozonation and wet rinsing for malodours. Ozone was injected into the rinse liquid, which was sprayed from nozzles using an injector. The study showed that ozone oxidation could remove hydrogen sulphide with up to 99% efficiency [103]. Another highly effective process that provides a high percentage of organic compound removal is thermal neutralisation. This uses the combustion of odorants in a stream of oxygen or air, where the structure of the compound is destroyed, thus eliminating its aromatic properties and so the malodorous substances. Unfortunately, this is an expensive process [101]. Mixed methods (adsorption + combustion) can be used to reduce the high cost of this process [100,104].

#### 6.2.5. Neutralization and Masking

**Figure 13:**
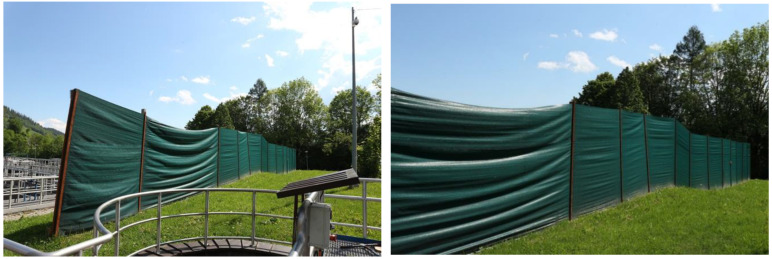
View of the shield fabric barrier located at the Zakopane WWTP (own photos).

In the case of surface emitters at wastewater treatment plants, or where there is no possibility of capturing polluted air, three methods of dealing with odour nuisance can be distinguished. The first is masking—which involves replacing a nuisance odour with a more pleasant odour [52]. The second method is fogging, which involves the use of compounds that exhibit neutralising properties. These include aldehydes and ketones [89]. However, studies have shown that the use of masking and neutralising agents, despite the reduction of odour nuisance (hedonically), can increase the odour concentration of emissions even above permissible values [105]. The third method is the use of shield mats, which are spread around the surface emitter. The use of a barrier about 2–2.5 m high made of a cover fabric (cover mat) of at least 300 g/m^2^ guarantees effective protection against strong wind gusts and, consequently, against the spread of malodorous gases and bioaerosols. Figure 13 shows an example of a shield fabric barrier at a wastewater treatment plant in Zakopane (Poland).

It should be noted that reduction of odour nuisance can be achieved by preventing odorous substances by intervening in the technological processes of already existing emission sources and making any possible technological changes, and taking into account odour emissions at the stage of designing such facilities [106].

In summary, the odour nuisance of wastewater treatment facilities can be reduced in two ways. The first way is to prevent odorants and release them through evaporation into the air. The second direction is based on collecting and treating odorants emitted at treatment plants. Particularly in wastewater treatment plants, the application of a prevention plan in the pre-treatment of wastewater and the reduction of odour-causing compounds at individual wastewater treatment facilities are considered preventive measures for odorant emissions [2]. With the above methods, the control of odour emissions from wastewater treatment plants can be achieved; however, such facilities, in order to effectively manage odour emissions, should be required to implement an odour management plan.

## 7. Conclusions

Wastewater treatment plants are undoubtedly a source of malodorous substances, which, even in low concentrations, have a negative impact on human health, causing, e.g., irritation of the throat and eyes, nausea, cough, moreover in high concentrations can lead to death. Referring to the analysis of the data included in this manuscript, the following conclusions have been formulated, which may constitute recommendations for the implementation of good practices to reduce the emission of substances hazardous to human health from wastewater treatment plants:

- Emission of odorous substances from wastewater treatment plants should be taken into account at the design stage of such a facility, by including appropriate solutions in the technological line (e.g., hermetisation of devices, purification of air taken from devices), which will reduce the impact of these substances on people and the environment. In many wastewater treatment plants that are struggling with the problem of odour nuisance, such solutions are introduced at the stage of operation, which is obviously a beneficial action. Nevertheless, they should be an indispensable element of the wastewater treatment process at the design stage.
- The operator of each wastewater treatment plant should take steps to determine the community reaction (CR) on this facility, which can be accomplished by collecting and analysing complaints from local residents, as well as conducting a surveys among these residents. It should be highlighted that the results of such surveys will also allow the operator to determine the range of impact of the facility, and this information can be the basis for the application of solutions which reduce and eliminate the impact of odorous substances.
- Despite the lack of direct legal regulations in Poland (and in many European Union countries) and the lack of the need to meet the requirements for the emission of odorous substances from the wastewater treatment plant, operators should show initiative to systematically perform such measurements, because the presence of certain substances in the air creates a risk to human health (HI > 1). It is worth pointing out that many substances included in the volatile organic compounds exhibit carcinogenic properties, which also confirms the validity of monitoring the presence of these substances in the air in the range of treatment plant.
- A paradigm shift should be pursued that odour emission is a local problem. Of course, the perception of odours is an individual matter; however, the unfavourable impact of malodorous substances on the human body is confirmed and indisputable. The issue of reducing the emission of odorous substances should be considered with the use of unambiguous legal regulations (in many countries, internal regulations regarding the prevention of odour nuisance have no legal force) and control methods with a confirmed degree of reliability of the obtained results.

## Figures and Tables

**Table 1:** Odour characteristics of chemical compounds found at wastewater treatment facilities, along with the degree of nuisance (own elaboration based on [29,30,31]).

Chemical Compounds	Mixtures of Chemical Compounds	Odour Characteristics	Characteristics Depending on Concentration with Indication of Possible Influence on Humans TD */LTEL **/STEL *** [mg/m^3^]	Degree of Nuisance
Sulphur containing compounds	Hydrogen sulphide	rotten eggs	0.14/7/14	High
	Methyl mercaptan (Methanethiol)	stinky, rotten cabbage, radish	0.0042/1/2	High
	Benzyl mercaptan	stinky, strong, disgusting	n.d.	High
	Butyl mercaptan (Butano-1-thiol)	rotten cabbage, skunk secretion, mustard	0.001–0.002/1/2	High
	Ethyl mercaptan (Ethanethiol)	stinky, skunk secretion, garlic	0.0001/1/2	High
	Allyl mercaptan (2-Propentiol)	stink, garlic, coffee	n.d.	High
	Diethyl sulphide	rotten vegetables, garlic, stinky	n.d.	High
	Dimethyl sulphide	rotten vegetables, cabbage, turnips, stinky	n.d.	High
	Dimethyl disulphide	stinky, disgusting	0.003–0.014/2.5/5	High
Nitrogen containing compounds	Ammonia	suffocating, pungent, irritating, ammoniacal	>0.4/14/28	Medium
	Methylamine	ammoniacal, fishy	0.03/5/15	Low
	Dimethylamine	ammoniacal, fishy	n.d./3/9	High
	Trimethylamine	ammoniacal, fishy	2/4.9/12.5	High
	Pyridine	faint, sweetish, unpleasant	0.04–14/5/n.d.	Medium
Other organic compounds	Indol	rotten proteins, faeces, faeces	n.d.	High
	Skatol	faeces, faeces	n.d.	High
Volatile fatty acids	Butyric acid	rancid butter, sweat	n.d.	High
	Propionic acid	pungent, rancid, irritating	0.084–60/30/45	Medium
	Acetic acid	pungent, acetic	0.5–2.5/25/50	Medium
Ketones	Acetone	sweet, musty, fruity, ethereal	484–968/600/1800	Medium
	Butanone	pungent, minty, similar to acetone	n.d.	Low

* TD—Threshold of Detection, ** LTEL—Long-Term Exposure Limit, *** STEL—Short-Term Exposure Limit, n.d.—no data.

**Table 2:** Impact of selected odorants on humans and the environment (own elaboration).

Chemical Compound	Impact on Humans	Impact on the Environment	Ref.
Hydrogen sulphide	respiratory failure, convulsions, hypotension, cardiac arrhythmia, nervous system impairment, lacrimation, death	in animals: respiratory problems, nervous system paralysis, death; causes acid rain in effect, lowering the pH of soil and water; water soluble—can travel long distances	[33,34]
Methyl mercaptan	watery eyes, cough, headache, scratchy throat, nausea, shortness of breath, convulsions, cyanosis, pulmonary oedema, liver and kidney damage, cardiac arrhythmia, loss of consciousness, narcotic effects	air pollution, flammable, aquatic pollution, toxic to aquatic organisms, damage to embryos or foetuses	[30,35]
Ammonia	corrosive effect, pain, tearing, swelling and redness of the eyes, cough, sore throat, salivation, nausea, vomiting, pain behind the sternum, shortness of breath, respiratory arrest, fibrosis of the lung tissue with severe respiratory failure	increased nitrogen deposition—degradation of sensitive ecosystems, formation of atmospheric aerosols, reduced visibility, impact on radiation balance, contribution to greenhouse gases, contribution to global warming, eutrophication	[30,36,37]
Ethyl mercaptan	nervous system impairment, convulsions, respiratory impairment, tearing and eye pain, headache, cough, diarrhoea	data not available	[38]
Volatile fatty acids	caustic effect, pain tearing and redness of the eyes, sore throat, cough, shortness of breath, bronchospasm, ulcers, visual disturbances, risk of corneal damage, vomiting, abdominal pain, possible bleeding from the gastrointestinal tract	acute and chronic toxicity to aquatic organisms, the ability to bioaccumulate in organisms, cause pH changes in water resulting in hypoxia of reservoirs	[29,30]
Dimethyl disulphide	cough, eye pain, photophobia, vomiting, abdominal pain, iron disorders, haemolytic anaemia, allergic dermatitis, headache	in animals: convulsions, coma, liver damage, haemolytic anaemia	[30]

**Table 3:** Selected legal regulations in force in Poland indirectly concerning the problem of odour nuisance (own elaboration based on [74,75]).

Legal Regulation	Content to Reduce/Eliminate Odour Nuisance
Act of 27 April 2001. Environmental Protection Law (Journal of Laws of 2001, item 519)	indication of reference values for individual substances in the air and activities whose conduct causes deterioration of the environment, which authorizes the issuance of a decision to suspend such activities
Act of 23 April 1964 Civil Code (Journal of Laws of 2017, item 459, as amended)	indicating that the owner of a building should refrain from activities that would interfere with the use of neighbouring properties beyond the average
Regulation of the Minister of Infrastructure of 12 April 2002 on the technical conditions to be met by buildings and their location (Journal of Laws of 2015, item 1422), Article 12, paragraph 6	indications regarding the location of a livestock building or an outbuilding on a plot in relation to a residential building, a collective residence building or a public utility building existing on an adjacent building plot
Act of 14 December 2012 on waste (Journal of Laws of 2013, item 21, as amended)	tightening regulations on landfills, waste incineration or co-incineration plants, waste transportation, and the use of municipal sewage sludge
Regulation of the Minister of Environment of 26 January 2010 on reference values for certain substances in the air (Journal of Laws of 2010 No. 16, item 87)	the determination of reference values, due to the need for health protection, for 167 substances or groups of substances, including odoriferous substances (ammonia, mercaptans, hydrogen sulphide or dimotylein)
Regulation of the Minister of Environment of 27 August 2014 on the types of installations that may cause significant pollution of individual natural elements or the environment as a whole (Journal of Laws of 2014, item 1169)	specification of the types of installations that may cause significant pollution of individual natural elements or the environment as a whole

**Table 4:** Legislation with reference to the resulting odours with the scope of its impact—recognition for different European countries (own elaboration based on [78,79,83,84,85,86,87]).

Country	Law	Scope of Influence
Netherlands	Based on Directive 2010/75/EU of the European Parliament and of the Council Netherlands Emission Guidelines (NeR)	All sectors covered by Directive 2010/75/EU of the European Parliament and of the Council. Mainly the animal husbandry sector
France	Based on Directive 2010/75/EU of the European Parliament and of the Council Ordinance of February 12, 2003 on animal by-product processing plants Ordinance of April 22, 2008 on composting plants	All sectors covered by Directive 2010/75/EU of the European Parliament and of the Council. Plants for processing animal by-products Composting plants Food and beverage processing
Germany	Air Protection Act Technical Instruction on Air Quality Control	List of substances and their emission limits Prevention of odour emissions from various technologies Determination of the distance of facilities from buildings
Czech Republic	Government Regulation No. 353/2002, establishing emission limits and other operating conditions for other stationary sources of air pollution, as amended	Agricultural sector
Hungary	No regulation, only calculations of odour impact distances are used, and the probability of exceedance (2%) should be used for the threshold range of odour concentration: 3 ou_E_/m^3^ < CT < 5 ou_E_/m^3^, where CT is the threshold concentration	Animal farms Solid waste landfills
Italy	No national regulations, only at the regional level	Composting plants

## Data Availability

The data used in this manuscript are not publicly available. However, they may be made available on the basis of an appropriate request addressed to the Chief Inspectorate of Environmental Protection in Poland (e-mail: sekretariatdp@gios.gov.pl) and the Department of Water and Wastewater Management in Trzebownisko (e-mail: Biuro@zgwstrzebownisko.pl).

## Abbreviations

NH_3_	Ammonia
BNR	Biological Nutrient Removal
CaO	Calcium oxide (Quick Lime)
Ca(OH)_2_	Calcium Hydroxide (Hydrated Lime)
CIEP	Chief Inspectorate of Environmental Protection
H_2_S	Hydrogen Sulphide
HI	Risk Assessment
LTEL (NDS in Poland)	Long-Term Exposure Limit
MBR	Membrane Bioreactor
MBBR	Moving Bed Biofilm Reactor
PAH	Polycyclic Aromatic Hydrocarbons
PCB	Polychlorinated Biphenyls
PIEP	Provincial Inspectorate of Environmental Protection
STEL (NDSCh in Poland)	Short-Term Exposure Limit
WWTP	Wastewater Treatment Plant
VOCs	Volatile Organic Compounds
